# Energy-efficient production of vaccine protein against porcine edema disease from transgenic lettuce (*Lactuca sativa* L.)

**DOI:** 10.1038/s41598-022-19491-z

**Published:** 2022-09-24

**Authors:** Asuka Yokoyama, Seika Oiwa, Takeshi Matsui, Kazutoshi Sawada, Yasushi Tasaka, Takeshi Matsumura

**Affiliations:** 1 Innovation Strategy and Carbon Neutral Transformation Department, Idemitsu Kosan Co., Ltd., 1-2-1, Otemachi, Chiyoda-Ku, Tokyo, Japan; 2 Advanced Technology Research Laboratories, Idemitsu Kosan Co., Ltd., 1280 Kamiizumi, Sodegaura-Shi, Chiba, 299-0293 Japan; 3 Bioproduction Research Institute, National Institute of Advanced Industrial Science and Technology, Sapporo, Hokkaido 062-8517 Japan

**Keywords:** Biotechnology, Plant sciences

## Abstract

The development of functional protein production systems using transgenic plants as hosts has been rapidly progressing in recent years. Lettuce (*Lactuca sativa* L.) has been studied as one such host, and it has been reported that the biomass of lettuce per area and target protein expression level can be increased by optimizing the cultivation conditions. Therefore, we investigated methods to minimize the input light energy per target protein to reduce production costs. Herein, we examined the yield of a nontoxic B subunit of Stx2e (Stx2eB) from transgenic lettuce under various cultivation conditions. Stx2eB acts as a vaccine against swine edema disease. The effects of photon flux densities (PPFDs), photoperiod, and light source on Stx2eB production were examined and the findings suggested that 400 μmol m^−2^ s^−1^, 24 h, and white LED lamps, respectively, contributed to energy-efficient Stx2eB production. In addition, Stx2eB was produced 1.4 times more efficiently per unit area time using a high plant density (228.5 plants m^−2^) than a common density (30.4 plants m^−2^). The findings of the present study can facilitate the development of energy-efficient and low-cost production processes for vaccine protein production, considering temporal and spatial perspectives.

## Introduction

Biopharmaceutical production based on *Escherichia coli,* yeast, insect, or mammalian cells is well established. Since the late 1980s, studies involving plant hosts have considerably advanced, with some practical applications^1^. The development of plant-made pharmaceuticals (PMPs) in closed production systems has numerous advantages, including rapid and efficient plant growth due to optimized conditions, low-cost and high-scale production, and low risk of contamination with human pathogens or toxins^2^. Transient expression systems using tobacco have been explored for the development of therapeutic drugs for the Ebola virus^3,4^, rotavirus vaccine proteins^5^, influenza vaccine^6^, and the COVID-19 vaccine^7^, with considerable progress being made. Recently, a techno-economic model and recombinant protein productivity per unit area time have been examined in the context of the business profitability of PMPs^8–10^.

A key advantage of PMP production is that by using edible plants as hosts, the plant itself can be used as the drug substance, without undergoing extraction and purification^11–13^, thereby making the resulting drugs relatively inexpensive since the costs for extraction and purification are omitted^2^. The approach also has several advantages in the production of livestock drugs for which vaccination is desired in the field. The drugs can be produced in bulk on site, transported and stored at lower cost and without refrigeration, and do not require injections, thereby removing the need for trained medical staff^14^. PMP production using edible plants has been studied since the late 1990s. The plants investigated include potato, tomato, lettuce, soybean, banana, rice, and strawberry, some of which are currently undergoing clinical trials^2,15–17^. Especially, using the transformation system of the chloroplast genome, recent advances include the production of coagulation factor IX using the cGMP hydroponic system^18^ and higher level of CTB-ACE2 expression levels in cGMP hydroponic facilities compared to greenhouse^19^. Additionally, the expression of the target protein was significantly improved by optimizing the growth conditions of lettuce, which further demonstrates the feasibility of clinical-grade plant biomass that can be prepared to meet the Food and Drug Administration (FDA) criteria^20^. Recently, the Phase I/II placebo-controlled, double-blind randomized study that of a therapeutic protein made in lettuce to prevent SARS-CoV-2 infection based on FDA approval, is in progress^21^. However, the design of a low-cost and energy-efficient upstream PMP production process has not yet been reported.

We have been developing a production system for a veterinary vaccine against swine edema disease (ED) using transgenic lettuce. ED is a bacterial disease caused by enterohemorrhagic *Escherichia coli* (STEC), which produces the Shiga toxin 2e (Stx2e). Transgenic lettuce can express the nontoxic B subunit (Stx2eB) as a vaccine antigen^22^, which can then be accumulated using a double repeated Stx2eB gene^23^. The recombinant Stxe2B has been confirmed to function as a vaccine^24^ and the oral administration of dried transgenic lettuce powder to pigs was sufficient to relieve the pathogenic symptoms of ED^25^. Furthermore, to address the issues of transgene-silencing and low productivity mature seeds obtained using the CaMV35S promoter, a LsUBQ promoter and a longer version of HSPT878 were used to produce thousands of seeds that had high levels of accumulated 2×Stxe2B without silencing^26^.

Lettuce is a popular host for producing therapeutic proteins because of its rapid growth and established large-scale production processes for the commercial food market, which is achieved using artificial light and vertical farming^27^. Many studies have evaluated the effectiveness of altering environmental conditions, such as light, CO_2_ concentration, temperature, humidity, and air velocity, to increase the produced biomass of lettuce^28,29^. While assessing the economic feasibility of production, the marketable leaf fresh weight maximum, as well as energy consumption, must be taken into account. From an economic standpoint, production should optimize productivity for food purposes, the cultivated area, and the maximum marketable leaf biomass per energy dose. However, during functional protein production, the cultivated area and maximum protein yield per unit of energy consumption are crucial. In addition to considering the cultivation conditions in commercial facilities, it is important for the commercial production of PMP to maximize the yield per area and reduce cultivation costs while minimizing the input energy per target protein. However, to the best of our knowledge, such a design of a low-cost and energy-efficient upstream production process has not yet been reported in the literature.

In this study, we aim to maximize the yield of Stx2eB as a vaccine against swine ED from transgenic lettuce. We investigated the effects of promoters, photosynthetic photon flux density (PPFD), photoperiod, and light source on Stx2eB accumulation. A combination of cultivation conditions that can provide energy-efficient vaccine yields has been presented.

## Results

**Figure 1:**
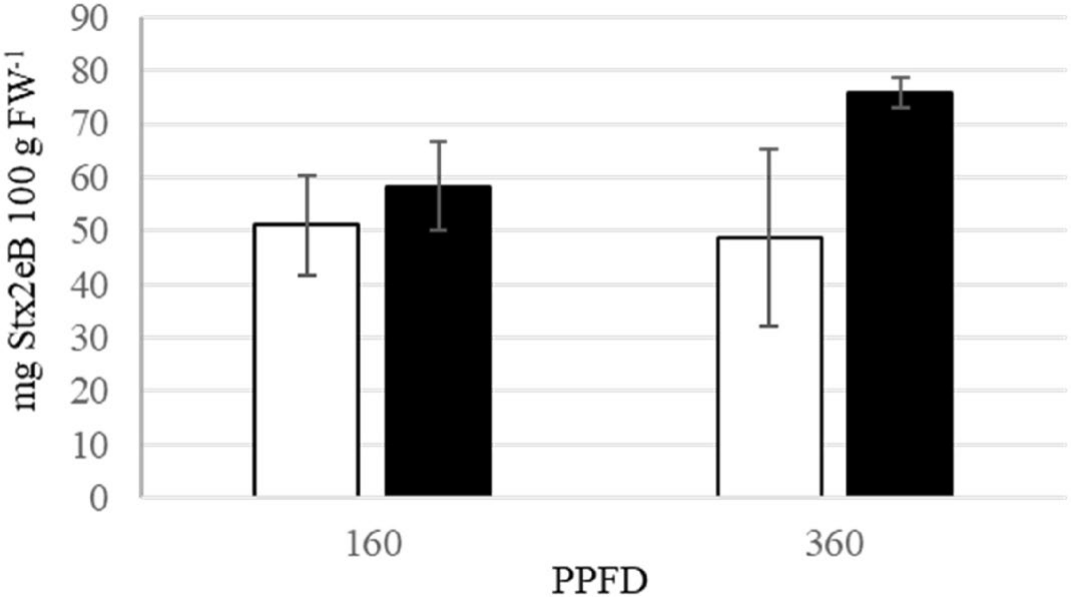
Effects of promoter on Stx2eB accumulation. Transgenic lettuce was grown under 160 and 360 μmol m^−2^ s^−1^ photosynthetic photon flux densities (PPFDs) for 35 days. White bars indicate 2BH (CaMV 35S pro.) and black bars indicate 2 BU (*Ls*UBQ pro). Error bars represent the standard error of the mean (n = 3). Comparisons of the means were performed using a Student’s t-test.

The effects of the different promoters on Stx2eB accumulation were investigated using 2BH and 2BU. No significant difference was observed between the effects of 160 and 360 μmol m^−2^ s^−1^ PPFD in 2BH, which is a Stx2eB gene driven by the CaMV 35S promoter (Fig. 1, white bars). The findings of the present study are in accordance with that of a previous study wherein no difference was observed in the concentration of Stx2eB in transgenic lettuce in which the Stx2eB gene was driven by the CaMV 35S promoter at 100–400 μmol m^−2^ s^−1^ PPFD^30^. However, in 2BU, the Stx2eB concentration was higher at a PPFD of 360 μmol m^−2^ s^−1^ than at that of 160 μmol m^−2^ s^−1^ (Fig. 1, black bars). In addition, the Stx2eB concentration was higher in 2BU than in 2BH under both the light conditions.

**Figure 2:**
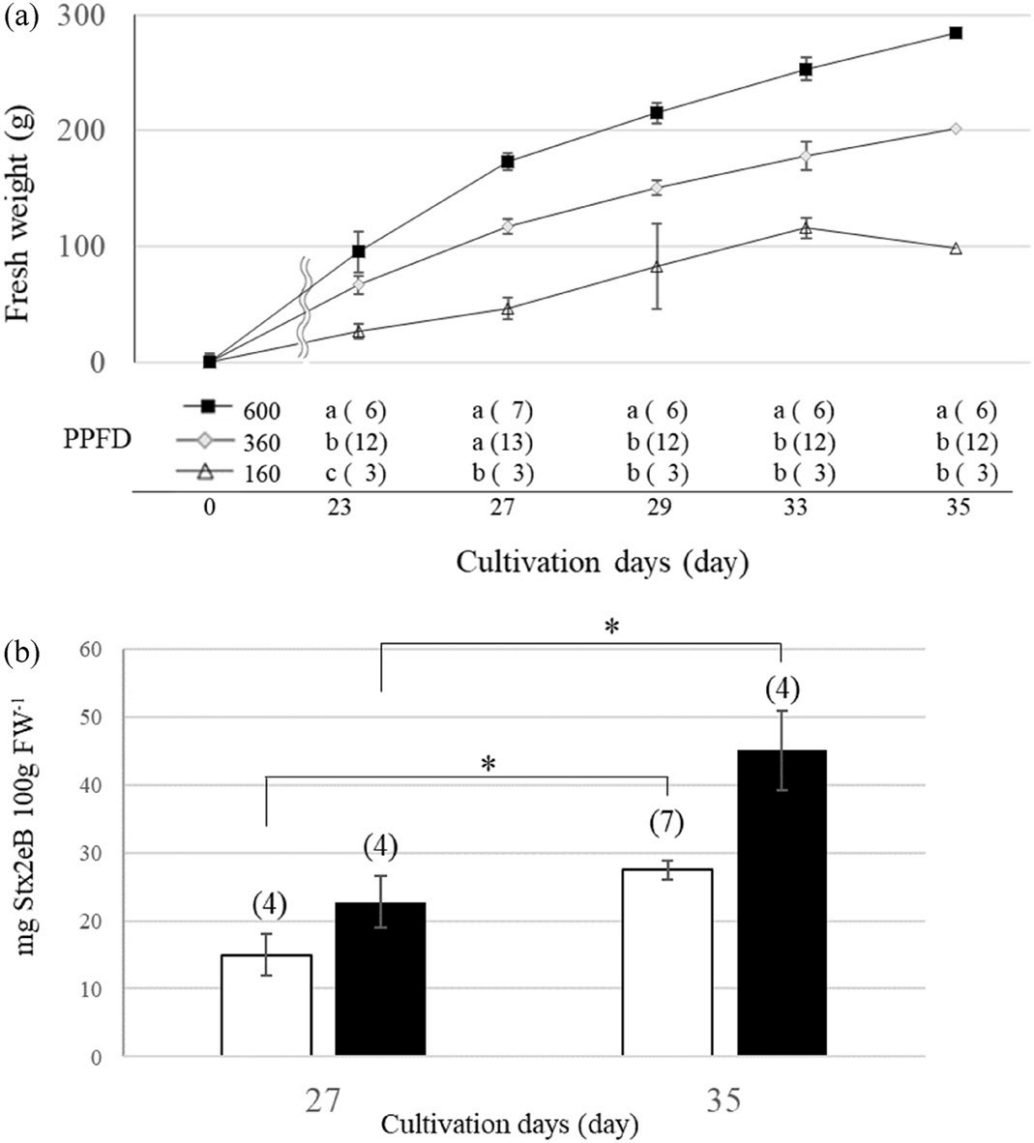
Effects of photosynthetic photon flux densities (PPFDs) on the fresh weight and Stx2eB concentration of 2 BU lettuce cultivated under a 16:8 h L:D photoperiod. (**a**) Fresh weight per plant. Different letters indicate significant differences between PPFD treatments at the *p* < *0.05* level according to a Tukey–Kramer test. Values in parentheses represent the number of plants. (**b**) Stx2eB concentration per 100-g fresh weight. White and black bars indicate 360 and 600 μmol m^−2^ s^−1^ PPFD, respectively. Asterisks indicate statistical differences using a Student’s t-test (*p < 0.05). Bars indicate the standard error of the mean. The values given in parentheses are the number of western blotting samples.

The effects of various PPFD levels on fresh weight and Stxe2B concentration were investigated using 2BU (Fig. 2). The lettuce fresh weight increased with PPFD. The average fresh weight at 600 μmol m^−2^ s^−1^ PPFD was ~ 284 g, which was 2.9 and 1.4 times higher than that at 160 and 360 μmol m^−2^ s^−1^ PPFD, respectively (Fig. 2a). At 600 μmol m^−2^ s^−1^ PPFD, the leaves were small, centered, dense, and strongly headed, and some brown outer leaves were observed (Supplementary Fig. 1). At 360 μmol m^−2^ s^−1^ PPFD, an intermediate phenotype (leaf spread, degree of heading) between those at 160 and 600 μmol m^−2^ s^−1^ was observed. The Stx2eB concentration approximately doubled between days 27 and 35 under both the light conditions (360 and 600 μmol m^−2^ s^−1^) (Fig. 2b). The Stxe2B concentration was higher at 600 μmol m^−2^ s^−1^ than at 360 μmol m^−2^ s^−1^ on the same cultivation day. The dose per lighting energy consumption was calculated (Table 1). The expression level of Stx2eB at a PPFD of 600 μmol m^−2^ s^−1^ was approximately 1.4–1.6 times higher than that at a PPFD of 360 μmol m^−2^ s^−1^ (7.3 mg gDW^−1^ or 8.0% Stx2eB in total soluble protein, Table 1F,H). Based on the number of plants per shelf, the doses per shelf were 2887 and 6324 for 360 and 600 μmol m^−2^ s^−1^, respectively (Table 1K). The lighting energy consumption per dose was estimated to be 1.5 and 1.1 kWh dose^−1^ at 360 and 600 μmol m^−2^ s^−1^, respectively (Table 1L). In conclusion, 600 μmol m^−2^ s^−1^ PPFD appears to be superior to 360 μmol m^−2^ s^−1^ PPFD in terms of the Stx2eB yield and lighting energy consumption.

**Table 1:** Analysis of dose per light energy consumption.

PPFD	(A)	(B)	(C)	(D)	(E)	(F)	(G)	(H)	(I)	(J)	(K)	(L)
	Lighting energy consumption per day	Lighting energy consumption per cultivation period	Fresh weight	Fresh weight per shelf	Moisture content	Stx2eB concentration	Total soluble protein (TSP)	% Stx2eB content/TSP	Stx2eB yield per plant	Stx2eB yield per shelf	Dose per shelf	Dose per lighting energy consumption
μmol m^−2^ s^−1^	kWh day^−1^	kWh period^−1^	g plant^−1^	kg/shelf (336plants)	%	mg gDW^−1^ ± SD	mg gDW^−1^ ± SD	%	mg plant^−1^	mg shelf^−1^	Dose	kWh dose^−1^
360	123.5	4323	201.6	67.7	5.4	5.4 ± 0.7	107.8 ± 30.9	5.0	59.3	19,924	2887	1.5
600	195	6825	284.5	95.6	6.3	7.3 ± 2.3	91.0 ± 26.6	8.0	129.9	43,634	6324	1.1

Mean lighting energy consumption per day (16 h photoperiod), the effects of photosynthetic photon flux densities (PPFDs) during 35 days of cultivation, and their combined effects on fresh weight (g plant^−1^), total soluble protein (mg DW^−1^) and Stx2eB concentration (mg g DW^−1^/mg 100 g FW^−1^) are shown. Stx2eB yield per shelf was calculated as 336 plants per shelf or a plant density of 30.5 plants m^−2^.

We cultivated transgenic lettuce under a 24 h photoperiod for 24 days and examined the effects of the combination of PPFD and photoperiod on lettuce fresh weight (Table 2). Fresh weight per unit lighting energy consumption under a 16 h photoperiod was calculated for 23 days. The fresh weight per unit lighting energy consumption with a daily light integral (DLI) of 5760 mol m^−2^ d^−1^ was 15.5 g kWh^−1^ at 16 h and 18.0 g kWh^−1^ at 24 h, and with a DLI of 9600 mol m^−2^ d^−1^, it was 14.3 g and 16.9 g kWh^−1^ at 16 h and 24 h, respectively (Table 2E). With equivalent DLI (DLI 5760 m^−2^ d^−1^; 16 h and 360 μmol m^−2^ s^−1^ PPFD, 24 h and 240 μmol m^−2^ s^−1^ PPFD: DLI 9600 m^−2^ d^−1^; 16 h and 600 μmol m^−2^ s^−1^ PPFD, 24 h and 400 μmol m^−2^ s^−1^ PPFD), a 24 h photoperiod produced a higher fresh weight yield with low PPFD, which was, however, approximately 20% higher than that produced in a 16 h photoperiod.

**Table 2:** Effect of photosynthetic photon flux densities (PPFDs) and photoperiod combination on lettuce biomass.

Light intensity	Day	Day light intensity	(A)	(B)	(C)	(D)	(E)	(F)
			Light energy consumption per day	Light energy consumption per cultivation period	Fresh weight	Biomass yield per shelf	Light energy consumption per biomass	Biomass per light energy consumption
(PPFD)	Length							
μmol/m^2^/s^1^	hour	DLI	kW/day	kW/period	g/plant	kg/shelf	g/kW	kW/kg
100	24	2400	44	1050	50	17	16.2	63
240		5760	74	1774	95	32	18.0	55
400		9600	113	2720	136	46	16.9	59
160	16	2560	35	813	27	9	11.1	91
360		5760	62	1420	67	22	15.5	63
600		9600	98	2243	95	32	14.3	70

Mean lighting energy consumption per day (24 h), effects of PPFD treatments during 24 days of the cultivation period, and their combined effects on fresh weight (g plant^−1^) were evaluated. Biomass yield per shelf was calculated at 336 plants per shelf. To compare the results with the same DLI, a 16-h photoperiod and a 23-d cultivation period were used to calculate the fresh weight per unit of lighting energy consumption. One dose was calculated as 6.9 mg, from a previous study^25^.

**Table 3:** Results of analysis of dose per unit lighting energy consumption with various light sources.

	Light intensity (PPFD)	(A)	(B)	(C)	(D)	(E)	(F)	(G)	(H)	(I)	(J)	(K)	(L)
	μmol/m^2^/s^1^	Lighting energy consumption per day	Lighting energy consumption per cultivation period	Fresh weight	Fresh weight per shelf	Moisture content	Stx2eB concentration	Total soluble protein (TSP)	% Stx2eB content/TSP	Stx2eB yield per plant	Stx2eB yield per shelf	Dose per shelf	Dose per lighting energy consumption
		kWh day^−1^	kWh period^−1^	g plant^−1^	kg/shelf (336plants)	%	mg gDW^−1^ ± SD	mg g DW^−1^ ± SD	%	mg plant^−1^	mg shelf^−1^	Dose	kWh Dose^−1^
Fluorescent light	400	98	3430	219.2	73.6	7.0	10.2 ± 1.4	81.0 ± 14.0	12.7	157.6	52,940	7672	0.4
LED	400	30	1050	207.2	69.6	6.2	10.1 ± 0.4	74.1 ± 15.1	13.6	129.1	43,367	6285	0.2
High rendering LED	400	40	1400	205.6	69.1	7.2	10.3 ± 1.0	70.3 ± 22.9	14.7	153.1	51,437	7455	0.2
Red green blue LED	400	160	5600	179.1	60.2	6.6	9.9 ± 1.6	76.3 ± 18.5	13.0	116.9	39,269	5691	1.0
Red blue LED	180	67	2345	180	60.5	6.1	9.5 ± 1.4	79.6 ± 13.5	12.0	105.1	35,325	5120	0.5

Mean lighting energy consumption per day (24 h photoperiod), photoperiod and photosynthetic photon flux densities (PPFDs) treatments during 35 days of cultivation, and their combined effects on fresh weight (g plant^−1^), total soluble protein (mg DW^−1^), and Stx2eB concentration (mg g DW^−1^/mg 100 g FW^−1^) are shown. Stx2eB yield per shelf was calculated at 336 plants per shelf. One dose was calculated as 6.9 mg, from a previous study^25^.

**Figure 3:**
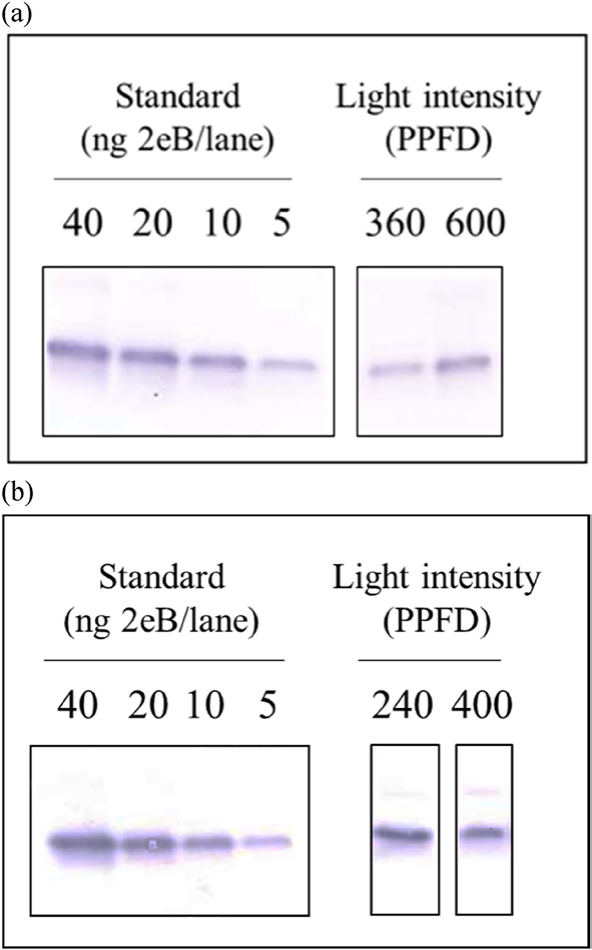
Western blotting assay results from different photoperiods and photosynthetic photon flux densities (PPFDs) under the same daily light integral (DLI). The combinations of photoperiod and PPFD for the same DLI were as follows. DLI 5,760 m^−2^ d^−1^: 16 h, 360 μmol m^−2^ s^−1^ PPFD; 24 h, 240 μmol m^−2^ s^−1^ PPFD. DLI 9600 m^−2^ d^−1^: 16 h, 600 μmol m^−2^ s^−1^ PPFD, 24 h, 400 μmol m^−2^ s^−1^ PPFD. (**a**) Antibody-reactive membrane loaded with extracts from transgenic lettuce cultivated in 16 h photoperiod for 23 days. Eleven of the samples were derived from the same experiment and blots were processed in parallel. (**b**) Same as (**a**) except cultivated under 24 h photoperiod for 24 days. All of the samples derive from the same experiment and the blots were processed in parallel.

No significant difference in Stx2eB concentration was observed between the 16 h and 24 h photoperiods (Fig. 3). Hence, the 24 h photoperiod appears to be superior to the 16 h in increasing the production of biomass without a reduction in Stx2eB concentration.

**Figure 4:**
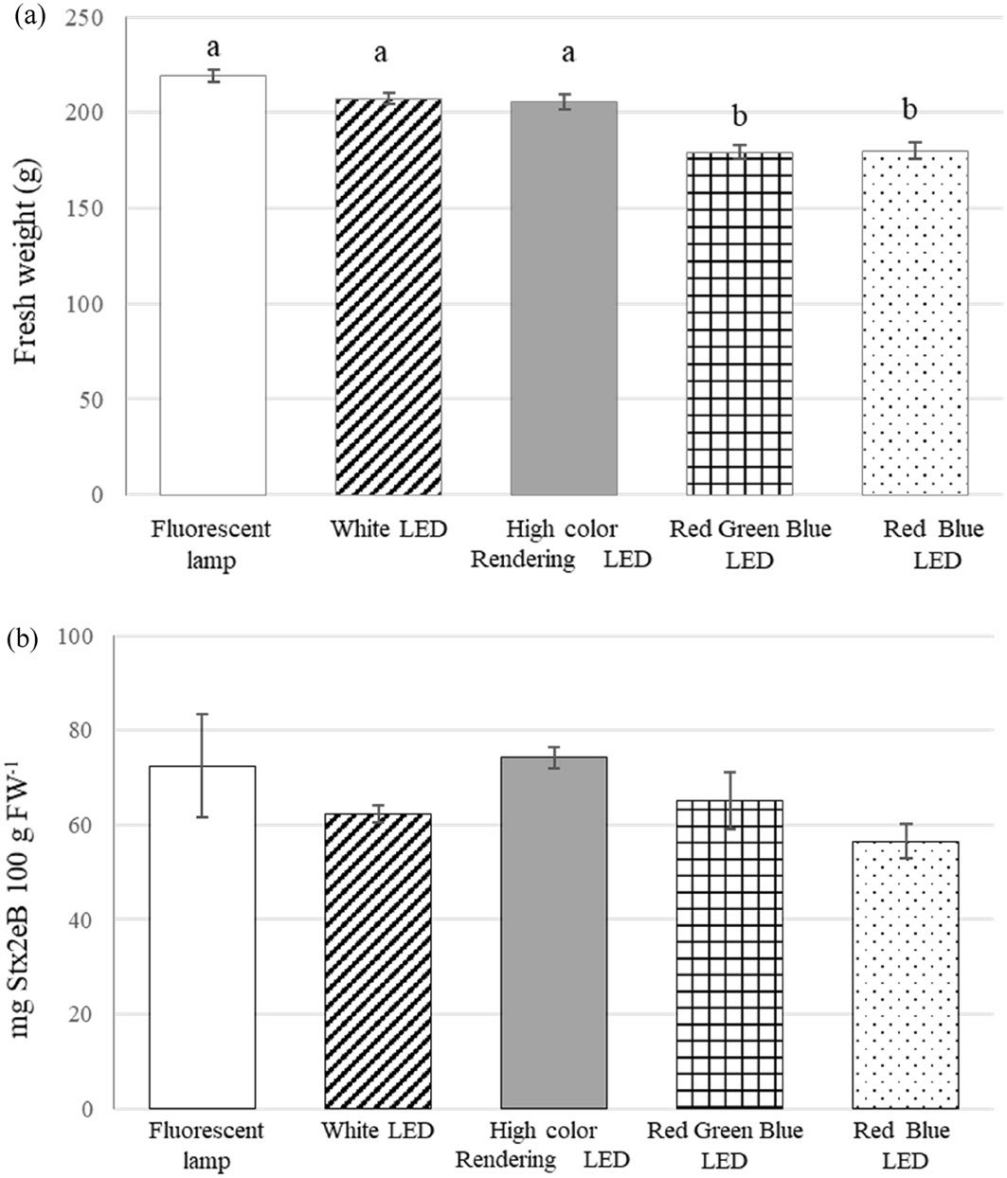
Effects of various light sources on the fresh weight and Stx2eB concentration of lettuce grown under 400 μmol m^−2^ s^−1^ photoperiod and photosynthetic photon flux densities (PPFDs) (fluorescent, white LED, high color-rendering LED, RGB LED lamps) and 180 μmol m^−2^ s^−1^ PPFD (RB LED lamp) 35 days after transplanting. (**a**) Fresh weight (n = 9–53). Different lowercase letters indicate significant differences at the *p* < 0.05 level according to a Tukey–Kramer test. (**b**) Stx2eB concentration per 100-g fresh weight (n = 3–8). Bars indicate the standard error.

The average fresh weights per plant after 35 days of cultivation were 219, 207, 206, 179, and 180 g for fluorescent, white LED, high color-rendering LED, Red Green Blue (RGB) LED, and Red Blue (RB) LED lamps, respectively (Fig. 4a). The fresh weight obtained under white lamps (fluorescent lamps [FLs], white LED, high color-rendering LED) was significantly greater than under the specific wavelength light sources (RGB LED and RB LED lamps). Although among the white light sources, the wavelength characteristics were different (Fig. 6), no significant differences in fresh weight results were observed between them. With respect to the effects of the light sources of specific wavelengths, no differences were observed between the RGB LED lamp at 400 μmol m^−2^ s^−1^ PPFD and RB LED lamp at 180 μmol m^−2^ s^−1^ PPFD. The RB LED lamp enhanced lettuce growth even under weak light, suggesting that the biomass yield was not affected by green light. As under the effect of strong PPFD, the fading, dark greening, strong curling, and hardening of leaves are also observed under the effect of strong light (Supplementary Fig. 2). No difference in the phenotype of lettuce was observed under the effects of the different light sources. Depending on the type of lamp used, the Stx2eB expression levels were 9.5–10.3 mg gDW^−1^ and 12–15% Stx2eB in total soluble protein (Table 3F,H); hence, no significant difference in the Stx2eB concentration was observed between during the usage of the different lamps (Fig. 4b). The lighting energy consumption per dose is shown in Table 3. The dose yield per energy, calculated as Stx2eB yield divided by lighting energy consumption (Table 3L), was 0.4, 0.2, 0.2, 1.0, and 0.5 for fluorescent, white LED, high color-rendering LED, RGB LED, and RB LED lamps, respectively. The white LED lamp and high color-rendering LED lamps were 50% more energy efficient than the other lamps.

**Figure 5:**
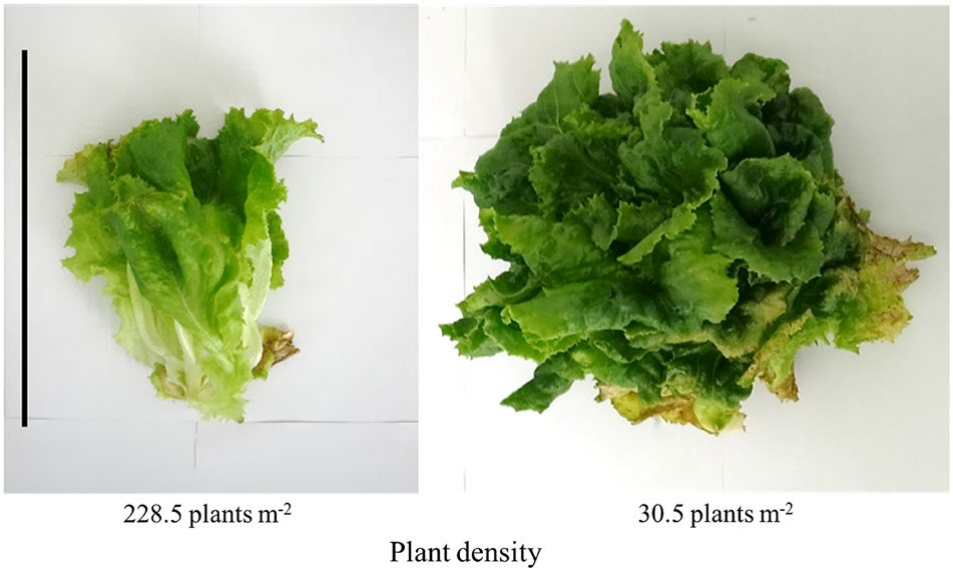
Effects of plant density on phenotype. Phenotype when lettuce was harvested. The seedlings were grown at 100 μmol m^−2^ s^−1^ photoperiod and photosynthetic photon flux densities (PPFDs) for 7 days after germination and were then transferred to cultivation panels. Transgenic lettuces were grown for 27 (228.4 plants m^−2^) or 35 days (30.5 plants m^−2^) after starting experimental fluorescent lighting conditions (24 h photoperiod, 400 μmol m^−2^ s^−1^ PPFD). The scale bar indicates 30 cm.

At a high density of 228.5 plants m^−2^, the panels became overcrowded on day 27, and consequently, were harvested. Plants at a density of 30.5 m^−2^ were harvested on day 35. At high densities, some leaves turned brown and became a cone, with no lateral spreading. At 30.5 plants m^−2^, some of the leaves hardened, strongly curled, faded, and became brown, which are also characteristics shown under high PPFD (Fig. 5). The expression levels of Stx2eB at 228.5 plant m^−2^ were approximately 1.4 times higher than that at 30.5 m^−2^ (13.1 mg g DW^−1^, Table 4D). Stx2eB content per total soluble protein (TSP) was similar at both densities (Table 4F). The Stx2eB yields per unit area multiplied by the number of plants were 5115.6 mg (741 doses) and 4695.8 mg (680 doses) at 228.5 plants m^−2^ and 30.4 plants m^−2^, respectively (Table 4G). Cultivation days were converted to months (27 days is 0.9 months and 35 days is 1.2 months; Table 4I) and the estimated Stx2eB productivity per unit area time was 567.1 at 30.5 plants m^−2^ and 823.8 at 228.5 plants m^−2^ (Table 4J).

**Table 4:** Results of analysis of Stx2eB productivity per unit area time at various plant densities used to evaluate the effects on fresh weight (g plant^−1^), total soluble protein (mg DW^−1^), and Stx2eB concentration (mg g DW^−1^/mg 100 g FW^−1^), area, and cultivation period.

Plant cultivation density	(A)	(B)	(C)	(D)	(E)	(F)	(G)	(H)	(I)	(J)
	Fresh weight	Fresh weight per area	Moisture content	Stx2eB concentration	Total soluble protein (TSP)	% Stx2eB content/TSP	Stx2eB yield per area	Dose per area	Cultivation period	Stx2eB productivity per unit area time
plants m^−2^	g plant^−1^	kg m^−2^	%	mg gDW^−1^ ± SD	mg g DW^−1^ ± SD	%	mg m^−2^	Dose m^−1^	month (days)	Dose m^−2^ month^−1^
228.5	35	7.9	4.9	13.1 ± 0.6	71.2 ± 10.1	18.5	5115.6	741.4	0.9 (27)	823.8
30.5	242	7.4	6.7	9.6 ± 2.6	58.2 ± 8.6	16.4	4695.8	680.6	1.2 (35)	567.1

One dose was calculated as 6.9 mg from a previous study^25^.

## Discussion

Using edible plants as a host for oral drug administration does not require extraction and purification, and therefore, has many advantages over functional protein production with tobacco^8^. Lettuce is particularly suitable as a host since large-scale production systems associated with it have already been established. Thus, various functional proteins, such as miraculin^31^, CTB-FIX^18^, S-hyIgA (secretary hybrid-IgG/IgA)^27^, Pro-IGF-1 (codon-optimized human insulin-like growth factor)^32^, and booster vaccine using poliovirus capsid protein^33^ have been produced using transgenic lettuce and cultivation tests were performed using the hydroponic system^18–20,33–36^. Recently, biomass yield and target protein concentration were dramatically increased by optimizing spacing, light intensity, nutrient solution, rock wool, and airflow^20^. The findings of these previous studies indicate the importance of studying the cultivation environment of lettuce to improve the yield of target proteins. Commercial production of functional proteins using edible plants requires high protein production with low initial and operating costs. Operating costs primarily comprise electricity and labor costs, with electricity mainly being for lighting^36^. Light conditions affect photosynthesis; therefore, the effects of light conditions, such as PPFD, photoperiod, and specific wavelengths, on fresh weight have been investigated. In the case of lettuce, the optimal PPFD, light quality, and photoperiod are 200 to 250 μmol m^−2^ s^−1^ PPFD, RB LED lamp supplemented with white LED lamps, and 16–18 h light d^−1^^28^. In addition, Zhang et al*.*^37^ reported that supplemental lighting delayed the senescence of outer leaves and decreased the incidence of dead or low-quality leaves, leading to improved marketable fresh leaf weight. However, the optimal environmental conditions for lettuce cultivation to obtain functional proteins are unclear. Hence, in this study, we examined the effects of a promoter, PPFD, photoperiod, light source, and plant density on Stx2eB yield and lighting energy consumption and determined the most effective conditions for the economic production of Stx2eB.

Previously, we constructed recombinant lettuce with 35S (2BH) or ubiquitin (2BU) promoters. The 2BH lettuce underwent transgene-silencing; however, the 2BU lettuce accumulated Stx2eB at high levels without transgene-silencing^26^. In this study, we investigated the combined effects of the cultivation environment and promoter used on Stx2eB accumulation. In 2BU, the Stx2eB concentration was higher under 360 μmol m^−2^ s^−1^ PPFD than under 160 μmol m^−2^ s^−1^ PPFD and was higher in 2BU than in 2BH under both the light conditions. Under conditions of high PPFD, it has been reported that ubiquitin and ubiquitination are involved in the labeling of damaged chloroplasts and contribute to protein degradation in senescing leaves^38,39^. Increased Stx2eB concentrations were also observed under high PPFD conditions (Fig. 1), as were numerous senescing leaves (Supplementary Fig. 1). Thus, strong light may have increased the expression level of the ubiquitin promoter.

The lettuce fresh weight under 600 μmol m^−2^ s^−1^ PPFD was significantly larger than that under 160 and 360 μmol m^−2^ s^−1^ PPFD (Fig. 2a). Chip burning, yellowing, and thickening of leaves were observed at 600 μmol m^−2^ s^−1^ PPFD; however, the Stx2eB concentration was higher at this PPFD than at lower PPFD levels. Hence, high PPFD potentially increases Stx2eB yields. The optimal range of PPFD for lettuce has been reported to be 200–250 μmol m^−2^ s^−1^^2^. Sago et al*.*^40^ also reported that increasing PPFD from 150 to 300 μmol m^−2^ s^−1^ increased chip burn. For lettuce production, increasing marketable leaves is important, and hence, the upper limit of PPFD, approximately 250 μmol m^−2^ s^−1^, is considered optimum for increasing fresh weight while suppressing leaf senescence. However, in this study involving lettuce cultivation as a functional protein production host, no relationship between the senescence of outer leaves and protein yield. The optimal PPFD condition for transgenic lettuce from the viewpoint of dose per unit lighting energy consumption was 600 μmol m^−2^ s^−1^ (Table 1).

Continuous illumination under low PPFD promotes the growth of leaf lettuce and enables growers to use less lighting energy than when using photoperiodic illumination with the same total DLI^41,42^. However, continuous light can also have a negative effect on the nitrate content of leaves and induce chip burn^28,41^. Hence, it has been suggested that 16–20 h light is an effective photoperiod to promote marketable leaf growth^43^. For the production of Stx2eB, our results indicate that high PPFD is efficient under a 16 h photoperiod but considering the energy consumption, it is better to obtain the Stx2eB yield with low PPFD. Therefore, we tested the hypothesis that the amount of biomass could be increased efficiently with a 24 h photoperiod. The fresh weight per lighting energy consumption in the 24 h photoperiod was 16.2–18.0 g kWh^−1^ under 100–400 μmol m^−2^ s^−1^ PPFD (Table 2). At 16 h, the fresh weight per lighting energy consumption was 11.1–15.5 g kWh^−1^ under 160 to 600 μmol m^−2^ s^−1^ PPFD. Hence, a 24 h photoperiod contributed to low lighting energy consumption and increased fresh weight. These results support those of previous studies that showed that when the DLI is the same, a 24 h photoperiod produces higher lettuce biomass than a 16 h photoperiod under low PPFD^44^. In addition, negative effects with the 24 h photoperiod, such as a decrease in Stx2eB concentration, were not confirmed. In summary, the optimal photoperiod condition for lettuce cultivation as a functional protein production host appears to be continuous light.

Currently, commercial plant factories for lettuce cultivation use FLs and LED lamps as light sources. Choosing the optimal light source is an important factor that affects initial costs, energy consumption, and yield. However, few studies have examined the optimum light source for recombinant protein production and reduced energy consumption. We analyzed the effects of a light source on fresh weight, Stx2eB concentration, and light energy consumption. In the present study, five different light sources were used and their effects on fresh weight, Stx2eB concentration, and lighting energy consumption were examined. The average fresh weight was significantly higher under white light sources than under light sources with specific wavelengths. It has been reported that RB lamps contribute to lettuce growth with low-energy consumption^45^ and green light increases plant growth by enhancing the photosynthetic rate of the leaves^46^. Shen et al*.* reported that aboveground of lettuce fresh weight under RB LED and WF lamps was 112 and 116 g, respectively in a closed plant chamber. Conversely, far less energy was consumed for RB LEDs than for WF lamps. As a result, RB LEDs produced early same aboveground biomass with far less energy consumption relative to WF lamps^44^. In the current study, the fresh weights obtained under white light sources and light sources with specific wavelengths were 211 g and 180 g, respectively. However, the growth-promoting effects of the light sources with specific wavelengths could not be confirmed. Furthermore, the growth-promoting effects of light sources with specific wavelengths may vary with the maturity level of lettuce. Hikosaka et al*.*^47^ reported that red light increased the expression level of human adiponectin under the control of a 35S promoter in transgenic strawberries; however, the underlying mechanism has not been elucidated. However, in contrast to the findings of our study, in the previously mentioned study, the light source used did not impact the Stx2eB concentration. Lighting energy consumption per cultivation period was 1050–5600 kWh depending on the light source. On comparing the lighting energy consumption per dose, the white LED and high color-rendering LED lamps were found to be optimal (0.2 kW dose^−1^), having half to one-fifth the energy use of the other lamps. Recently, it was reported that supplemental far-red light increased leaf expansion, consequently increasing biomass^48^. Therefore, further optimization of the spectra conditions during energy consumption is required.

Fujiuchi et al.^10^ emphasized the importance of improving the recombinant protein productivity per unit area time (unit: mg m^−2^ month^−1^) by examining the effect of different plant densities on the yield of recombinant hemagglutinin in a transient expression system. In this study, the effects of a common lettuce planting density (30.5 plants m^−2^) and a high plant density (228.5 plants m^−2^) were evaluated. At the high plant density, the cultivation panels became overcrowded, and the phenotype and size differed from those under the lower-density conditions. No difference in Stx2eB concentration per weight was observed between plant densities and there was no relationship between lettuce growth and Stx2eB concentration. The Stx2eB productivity was 822.0 g m^−2^ month^−1^ for high plant density and 581.8 g m^−2^ month^−1^ for common plant density, with Stx2eB being produced 1.4 times more efficiently under high-density conditions (Table 4). Okamura et al*.* reported the target protein concentration per g DW in recombinant lettuce under 22.2 plants m^−2^ or 222.2 plants m^−2^ conditions; the target protein concentration in the high plant density condition was half of that in the low plant density condition^30^. Conversely, in our experiment, the Stx2eB concentrations of high and low plant densities were 13.1 mg DW^−1^ and 9.6 mg DW^−1^, respectively, indicating that the concentration was higher in the high plant density conditions. As a result, the Stx2eB yield per area was greater under high-density conditions.

In this study, 400 μmol m^−2^ s^−1^ PPFD, 24 h photoperiod, white LED lamps, and high plant density conditions increased Stx2eB yield with low lighting energy consumption; hence, these factors represent the suitable cultivation conditions under which efficient Stx2eB yields can be obtained. These conditions differ from optimal lettuce cultivation conditions. Based on the findings presented above, cultivation for functional protein production is different from conventional crop cultivation, which aims to improve biomass yield, taste, nutritional content, and appearance. These findings have important implications for the low-cost and energy-saving design of upstream processes in edible PMP production systems.

## Methods

Expression of the Stx2eB gene in lettuce (*Lactuca sativa* L., cv. Green wave) is driven by the CaMV 35S (2BH) or UBQ (2 BU) promoters^26^. t_1_ and t_3_ seeds in 2BH and 2 BU, respectively, were used in this study. Lettuce seeds were sown in urethane sponges after sterilization by rinsing with 0.05% hypochlorous acid. The sponges were placed in a cultivation room at 23 °C, 100 m^−2^ s^−1^ PPFD, and 16:8 L:D photoperiod for 7 days. A week after sowing, the seedlings were transplanted to floating panels (59 × 89 cm) with a plant density of 48 plants per panel on a shelf (6600 mm W × 736 mm  D  × 1900 mm H, GFM Plant Shiki Bed 60 B, M Hydroponic Research CO, Ltd., Aichi, Japan) and were exposed to different PPFD conditions for 7 days after transplanting, unless otherwise described. During the cultivation period, the room temperature (22.5 °C), relative humidity (60% RH), and CO_2_ concentration (1000 μmol mol^−1^) were maintained. For the hydroponic nutrient solution, 1/2 OAT house A nutrient (OAT Agrio Co., Ltd, Tokyo, Japan) solution (pH 5.8, EC 1.8 dS m^−1^) was used.

**Figure 6:**
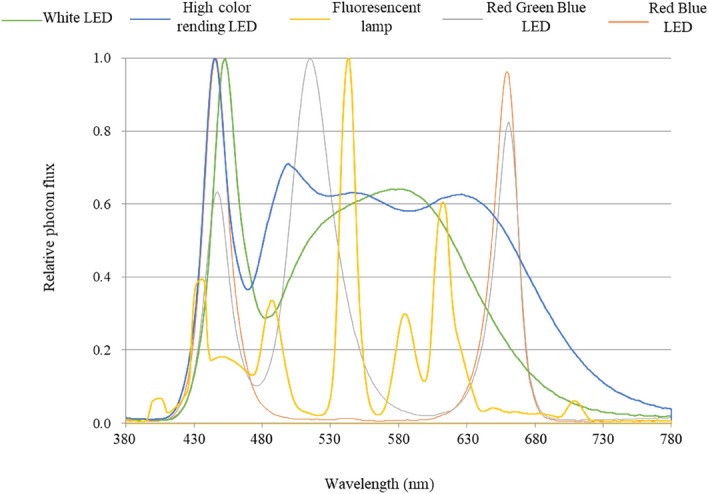
Light spectra of the light sources used for the cultivation of transgenic lettuce (*Lactuca sativa* L., cv. Green wave) transplants.

The light conditions in each experiment are described in Table 5. FLs (FHF32EX-N-H, Panasonic Corporation, Osaka, Japan) were used unless otherwise specified. The PPFD was established based on the intensity at the center of the cultivation panel, which was measured using a PPFD sensor (MQ200, Apogee Instruments Inc., Logan, UT, USA) and adjusting the number of tubes. White LED lamps (ECL-LE4EGN, Ecorica, Osaka, Japan), high color-rendering LED lamps (ECL-LE4EGN-L3A, Ecorica Inc.), and two types of special wavelength LED lamps (RGB LED and RB LED, Sekishin Group, Tokyo, Japan) were also used, which had PPFD values of 400 μmol m^−2^ s^−1^, except the RB LEDm whose PPFD was 180 μmol m^−2^ s^−1^. The PPFD of white LED lamps and high color-rendering LED lamps was adjusted for the number of tubes. The PPFD of the LED panel was adjusted in the same way as that of the FL, using a dimming knob attached to the panel. The light spectra shown in Fig. 6 were measured using MK350S (UPRtek, Miaoli, Taiwan).

**Table 5:** PPFD, photoperiod, and light source for each experiment in this study.

Experiment	Photoperiod (h)	PPFD (μmol m^−2^ s^−1^)	Light source
Expression levels of Stx2eB with 35S and UBQ promoters	16	160	FL
		360	
Effects of PPFD on fresh weight and Stxe2B concentration	16	160	FL
		360	
		600	
Effect of PPFD and photoperiod combination on lettuce yield	24	100	FL
		240	
		400	
Effects of light source on fresh weight and Stx2eB protein concentration and lighting energy consumption	24	400	FL
			White LED
			High color-rendering LED
			Red green blue LED
		180	Red blue LED
Effects of plant density on Stx2eB productivity per unit area time	24	400	FL

*FL* fluorescent lamp, *LED* light emitting diodes.

Lettuce shoots were harvested and the fresh weight was measured on day 35 (Experiment: Expression Levels of Stx2eB with 35S and UBQ Promoters); days 23, 27, 29, 33, and 35 (Experiment: Effects of PPFD on Fresh Weight and Stxe2B Concentration); day 24 (Experiment: Effects of PPFD and Photoperiod Combination on Lettuce Yield); day 35 (Experiment: Effects of Light Source on Fresh Weight, Stx2eB Protein Concentration, and Lighting Energy Consumption); and day 27 or 35 (Experiment: Effects of Plant Density on Stx2eB Productivity Per Unit Area Time). Sampling was conducted within the range of 80% plant density, which does not affect the amount of fresh weight per plant^30^. The total lettuce yield per shelf of 336 plants per shelf was 30.5 plants m^−2^ and of 2520 plants, 228.5 plants m^−2^. The harvested lettuce was frozen at − 80 °C with three plants in a bag. Frozen lettuce leaves were freeze-dried in a lyophilizer (FD-6BM-SQ, NIHON TECHNO SERVICE Co., Ltd., Ibaraki, Japan). The lyophilized leaves were ground in a blender (Wonder Blender, OSAKA CHEMICAL Co., Ltd, Osaka, Japan) at maximum speed for 10 s. The obtained powder was used for analysis. TSP was extracted from the dried powder by the TCA-Acetone method^30^. In brief, the dried protein precipitate was suspended in denaturation extraction buffer (6 M urea, 5 mM imidazole, 0.5 M sodium chloride, 20 mM Tris–HCL, pH 7.9), and the supernatant liquid was collected after centrifugation (16,000×*g* for 16 min at 4 °C). The TSP concentrations in clarified plant extracts were determined using a Protein Assay Kit II (Bio-Ra., Hercules, CA). The accumulation of Stx2eB protein was analyzed using semi-quantitative western blotting, as described previously^23^.

The lamps on the cultivation shelf were turned on for 3–5 days, and the energy consumption of the lighting was measured with an electricity meter directly connected to the cultivation shelf. After calculating the average value per day, the number of cultivation days was used to calculate the amount of lighting power consumed during the cultivation period. The dose per light energy consumption was calculated in accordance with our previous study, since the ED was relieved after three doses of 2.3 mg, the single dose was expected to be 6.9 mg^25^.

(Experiment: Expression levels of Stx2eB with 35S and UBQ promoters) 2BH and 2BU seeds were sown as described before. One week after sowing, seedlings were transplanted to floating panels (59 × 89 cm) with a plant density of 48 plants per panel and were exposed to either160 or 360 μmol m^−2^ s^−1^ PPFD and a 16-h photoperiod. One week after transplanting, nursery plants were transplanted to floating panels (59 × 89 cm) at a density of 30.5 plants m^−2^ and cultivated for 28 days (light exposure period was 35 days).

(Experiment: Effects of PPFD on fresh weight and Stxe2B Concentration) 2BU seedlings 7 days after sowing were transplanted as described before and were exposed to 160, 360, and 600 μmol m^−2^ s^−1^ PPFD, and a 16-h photoperiod. One week after transplanting, the nursery plants were again transplanted to 16 plants panel^−1^ (59 × 89 cm) at a density of 30.5 plants m^−2^ and cultivated for 16, 20, 22, 26, and 28 days (the light exposure periods were 23, 27, 29, 33, and 35 days). The accumulation of Stx2eB protein was analyzed using samples from 360 and 600 μmol m^−2^ s^−1^ PPFD.

(Experiment: Effects of PPFD and photoperiod Combination on lettuce yield) 2BU seedlings 7 days after sowing were transplanted as described before and were exposed to 100, 240, and 400 μmol m^−2^ s^−1^ PPFD, and a 24-h photoperiod. One week after transplanting, the nursery plants were again transplanted to 16 plants panel^−1^ (59 × 89 cm) at a density of 30.5 plants m^−2^ and cultivated for 17 days (light exposure period was 24 days).

(Experiment: Effects of light source on fresh weight, Stx2eB Protein concentration, and lighting energy Consumption) FL and white LED and high color-rendering LED lamps were tested under 400 μmol m^−2^ s^−1^ PPFD and 24-h photoperiod. To investigate the effects of a specific wavelength, a panel LED lamp with an adjustable RGB ratio was also tested under the same PPFD and photoperiod. Furthermore, the effects of green light were evaluated using an RB LED lamp, which removed only green from the RGB LED lamp and produced 180 μmol m^−2^ s^−1^ PPFD. All light source treatments were initiated after transplanting the seedlings, 7 days after sowing, and cultivated for 28 days (light exposure period was 35 days).

(Experiment: Effects of plant density on Stx2eB productivity per unit area time) 2BU seedlings were transplanted 7 days after sowing to 120 plants panel^−1^ (59 × 89 cm) at a density of 228.5 plants m^−2^ in high-density conditions and cultivated until they were overcrowded and harvested 27 days after starting light exposure. The reference condition was 30.5 plants m^−2^, and lettuce were harvested 35 days. The light condition was a FL, 400 μmol m^−2^ s^−1^ PPFD, and a 24-h photoperiod.

All of the experiments were conducted in accordance with the Cartagena Law Class 2 diffusion prevention measures.

## Supplementary Information

Supplementary Figures.

Supplementary Information 2.

Supplementary Information 3.

Supplementary Information 4.

Supplementary Information 5.

Supplementary Information 6.

Supplementary Information 7.

Supplementary Information 8.

Supplementary Information 9.

Supplementary Information 10.

## Data Availability

The data available from this article will be shared at the reasonable request to the corresponding author.
