# Supplementary material for: Energy-efficient production of vaccine protein against porcine edema disease from transgenic lettuce (Lactuca sativa L.)
Source: Sci Rep. 2022 Sep 24;12:15951. doi: 10.1038/s41598-022-19491-z (PMC9509315; doi:10.1038/s41598-022-19491-z)
Supplement: Supplementary file 1 — Supplementary Figures. [file 41598_2022_19491_MOESM1_ESM.docx]

**Supplementary Figure. S1** Effects of photosynthetic photon flux densities (PPFDs) on recombinant lettuce phenotype.


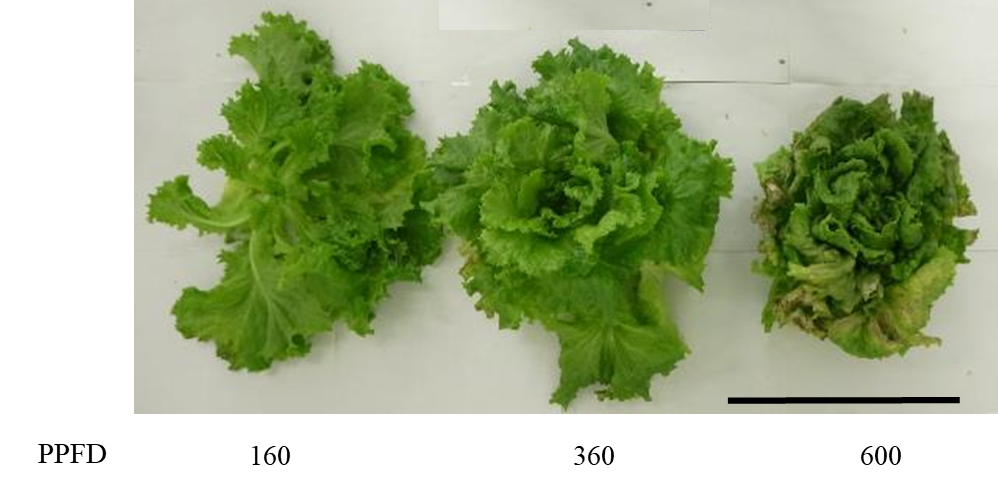


The lettuces were grown at various PPFD for 35 d with fluorescent lamps. Scale bar indicates 30 cm.

**Supplementary Figure. S2** Effects of light sources on recombinant lettuce phenotype.


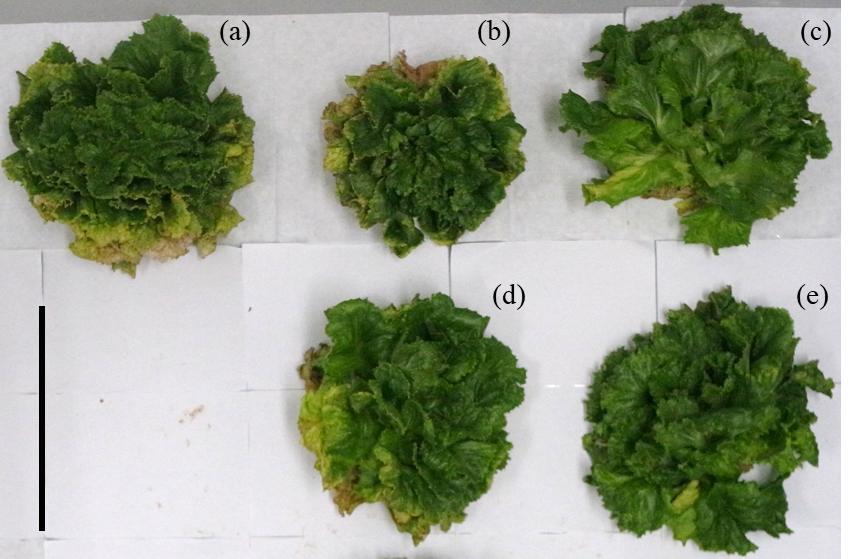


(a) Fluorescent light (b) High rendering LED (c) LED (d) Red green blue LED (e) Red blue LED. The lettuces were grown for 35 d. Scale bar indicates 30 cm.
