# Supplementary material for: Energy-efficient production of vaccine protein against porcine edema disease from transgenic lettuce (Lactuca sativa L.)
Source: Sci Rep. 2022 Sep 24;12:15951. doi: 10.1038/s41598-022-19491-z (PMC9509315; doi:10.1038/s41598-022-19491-z)
Supplement: Supplementary file 2 — Supplementary Information 2. [file 41598_2022_19491_MOESM2_ESM.docx]

**Supplementary Information about Figure 3.**

**Fig. 3** Western blotting assay results from different photoperiods and photosynthetic photon flux densities (PPFDs) under the same daily light integral (DLI).

1. (b)


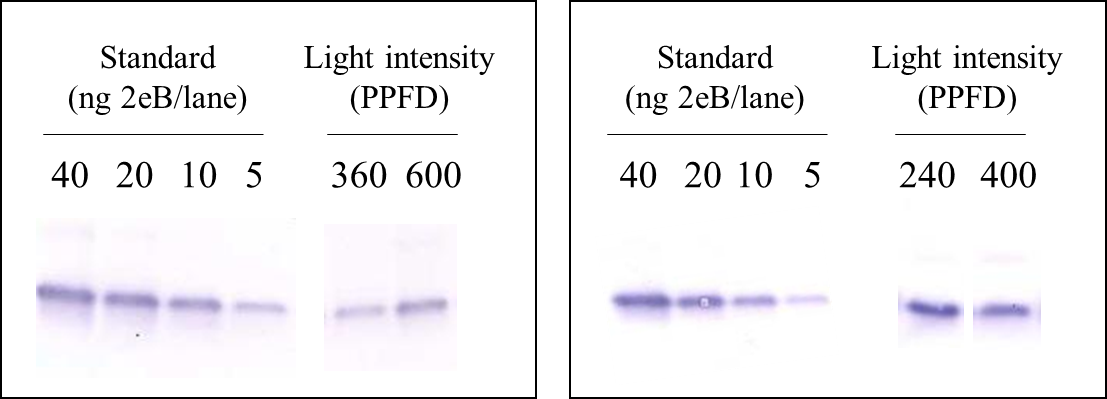


Combinations of photoperiod and PPFD for the same DLI were as follows. DLI 5,760 m^−2^ d^−1^: 16 h, 360 μmol m^−2^ s^−1^ PPFD; 24 h, 240 μmol m^−2^ s^−1^ PPFD. DLI 9,600 m^−2^ d^−1^: 16 h, 600 μmol m^−2^ s^−1^ PPFD, 24 h, 400 μmol m^−2^ s^−1^ PPFD. (**a**) Antibody-reactive membrane loaded with extracts from transgenic lettuce cultivated under 16 h photoperiod for 23 d. All of the samples derive from the same experiment and that blots were processed in parallel. (**b**) Same as (**a**) except cultivated under 24 h photoperiod for 24 d. All of the samples derive from the same experiment and that blots were processed in parallel.

**Supplementary Information about Figure 3.**

Figure 3 (a); Raw photo and Sample name
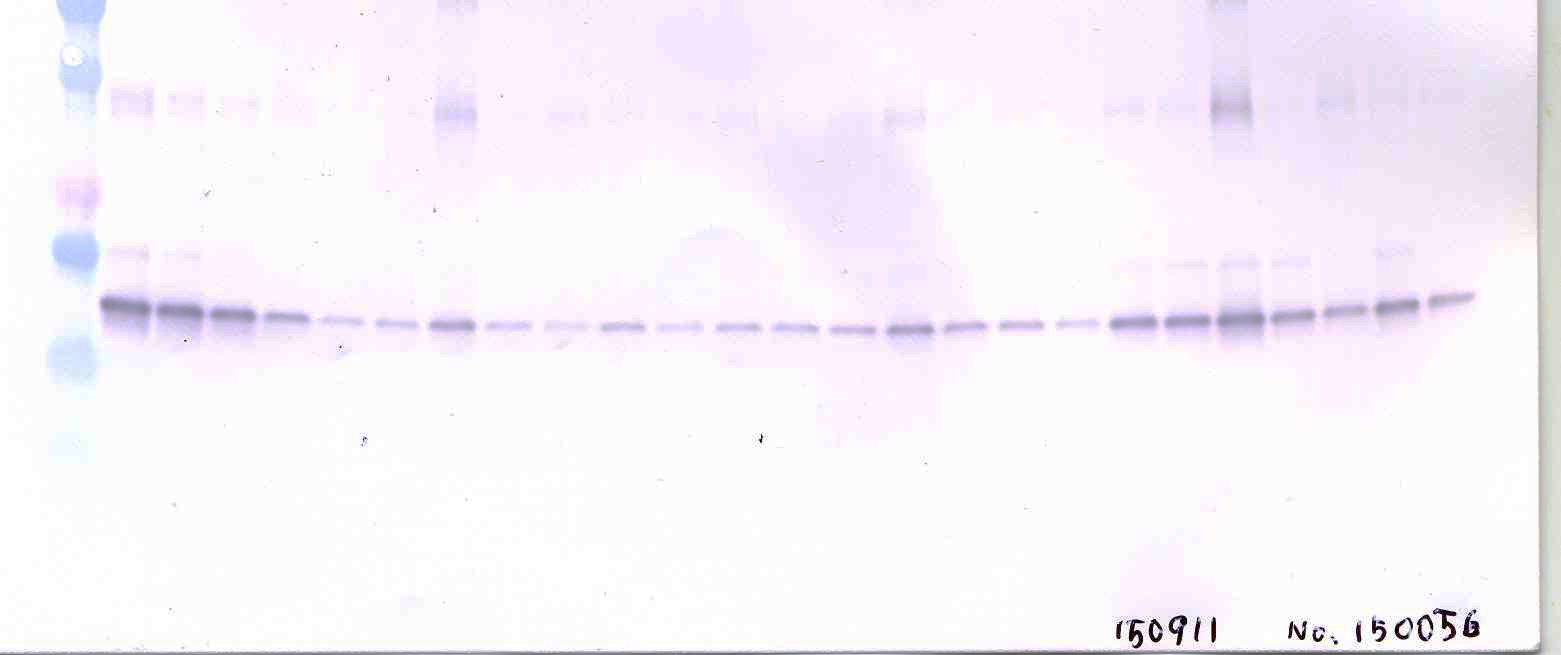


1 2 3 4 5 6 7 8 9 10 11 12 13 14 15 16 17 18 19 20 21 22 23 24 25

|  |  |  |  |
| --- | --- | --- | --- |
| Lane No. | Sample name |  |  |
| 1 | Standard (ng 2eB/lane) | 40 | the samples derive from here |
| 2 |  | 20 |  |
| 3 |  | 10 |  |
| 4 |  | 5 |  |
|  | PPFD | Dilution rate |  |
| 5 | 360 | 243 |  |
| 6 | 360 | 243 |  |
| 7 | 160 | 243 |  |
| 8 | 360 | 243 |  |
| 9 | 360 | 243 |  |
| 10 | 600 | 243 |  |
| 11 | 600 | 243 |  |
| 12 | 360 | 243 |  |
| 13 | 360 | 243 |  |
| 14 | 160 | 243 |  |
| 15 | 360 | 243 |  |
| 16 | 360 | 243 |  |
| 17 | 600 | 243 |  |
| 18 | 600 | 243 |  |
| 19 | 360 | 81 |  |
| 20 | 360 | 81 |  |
| 21 | 160 | 81 |  |
| 22 | 360 | 81 |  |
| 23 | 360 | 81 | the samples derive from here |
| 24 | 600 | 81 |  |
| 25 | 600 | 81 |  |

Figure 3 (b); Raw photo and Sample name


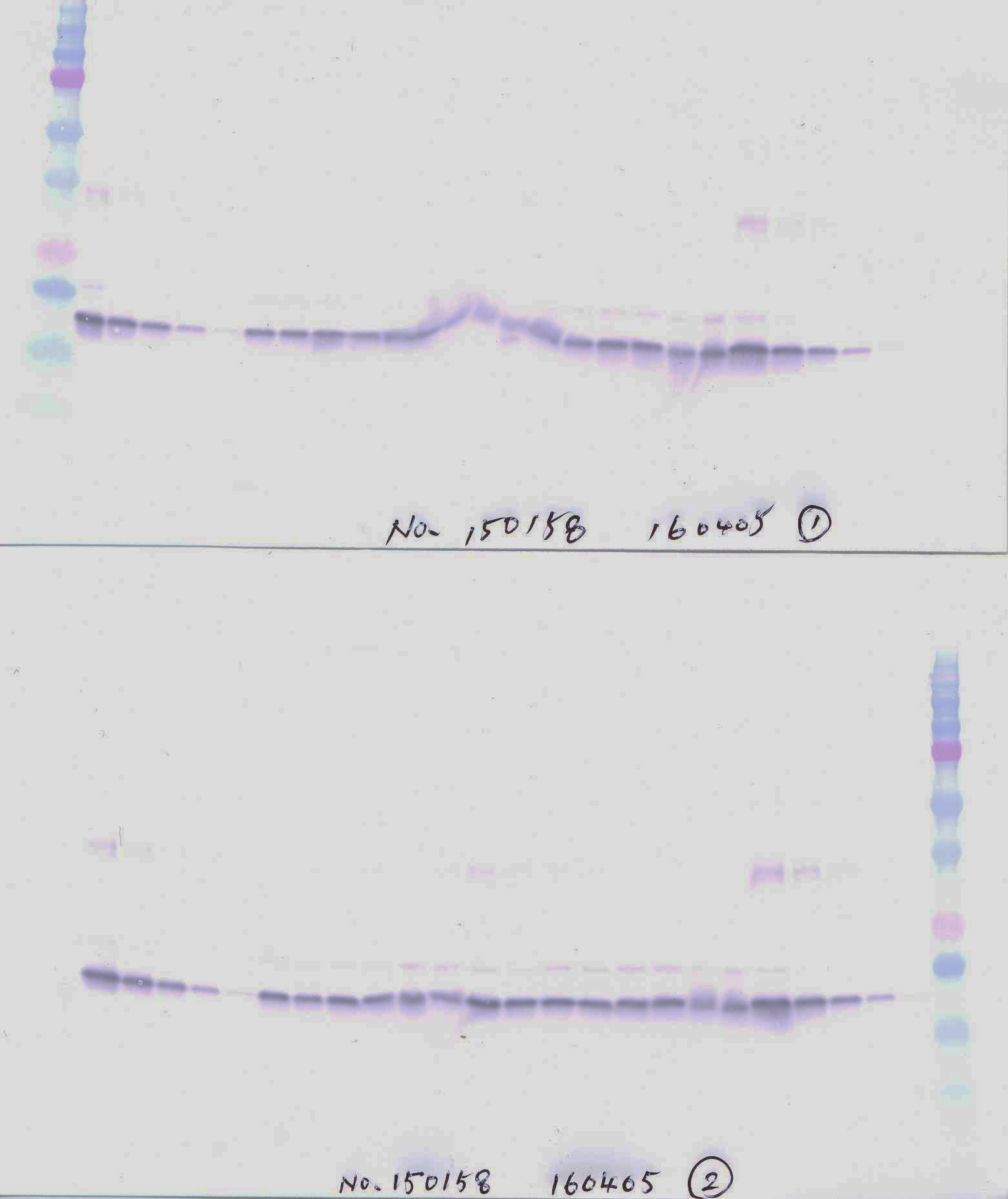


1 2 3 4 5 6 7 8 9 10 11 12 13 14 15 16 17 18 19 20 21 22 23

| lane No. | Sample name |  |  |
| --- | --- | --- | --- |
| 1 | Standard (ng 2eB/lane) | 40 | the samples derive from here |
| 2 |  | 20 |  |
| 3 |  | 10 |  |
| 4 |  | 5 |  |
| 5 |  | 2.5 |  |
|  | PPFD | Dilution rate |  |
| 6 | 240 | 81 |  |
| 7 | 100 | 81 |  |
| 8 | 400 | 81 |  |
| 9 | 240 | 81 |  |
| 10 | 500 | 81 |  |
| 11 | 400 | 81 |  |
| 12 | 240 | 81 | the samples derive from here |
| 13 | 100 | 81 |  |
| 14 | 400 | 81 |  |
| 15 | 240 | 81 |  |
| 16 | 500 | 81 |  |
| 17 | 400 | 81 | the samples derive from here |
| 18 | another experiment | 81 |  |
| 19 | another experiment | 81 |  |
|  |  |  |  |
| 20 | Standard (ng 2eB/lane) | 40 |  |
| 21 |  | 20 |  |
| 22 |  | 10 |  |
| 23 |  | 5 |  |

**Multiple exposure images**

**Pattern 1**

1. **(b)**


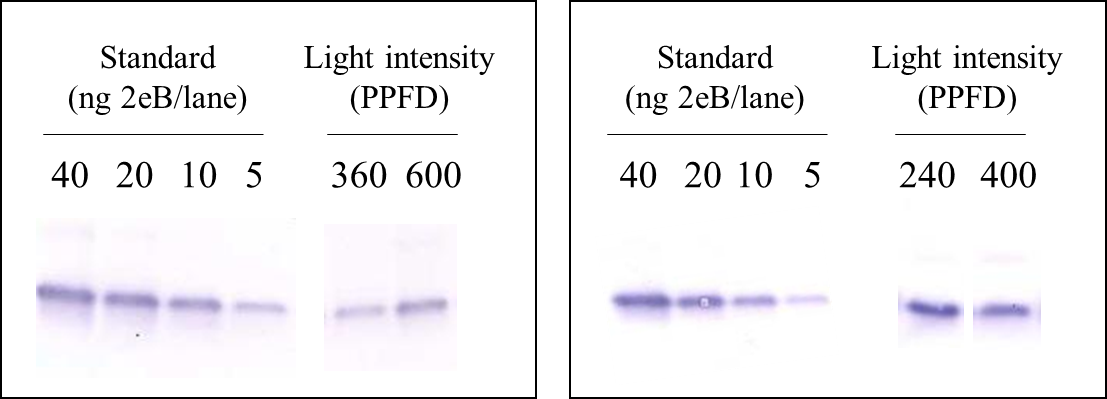


**Pattern 2**

1. **(b)**

**
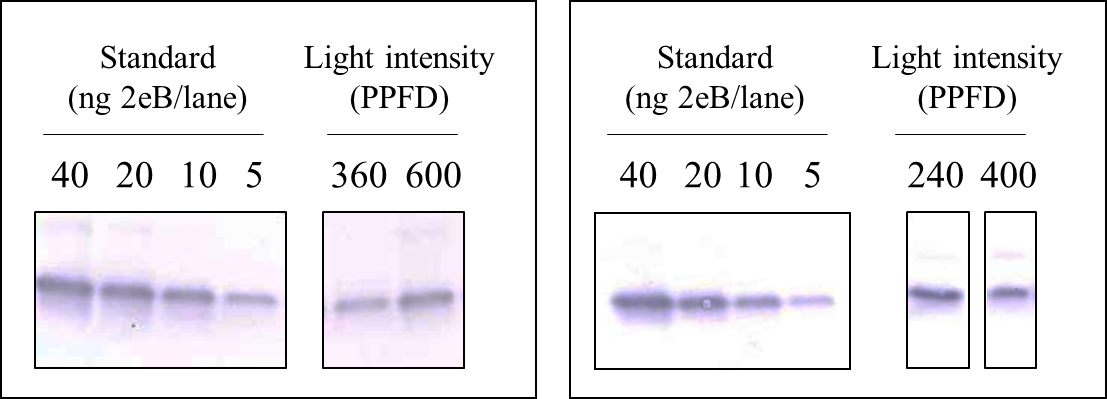
**

**Pattern 3**

**(a) (b)**

**
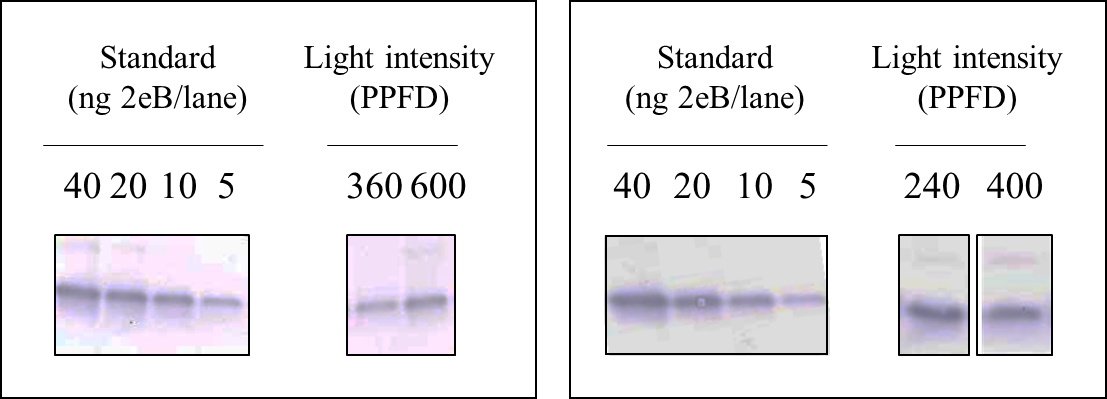
**
