# Supplementary figures and images for: Energy-efficient production of vaccine protein against porcine edema disease from transgenic lettuce (Lactuca sativa L.)
Source: Sci Rep. 2022 Sep 24;12:15951. doi: 10.1038/s41598-022-19491-z (PMC9509315; doi:10.1038/s41598-022-19491-z)

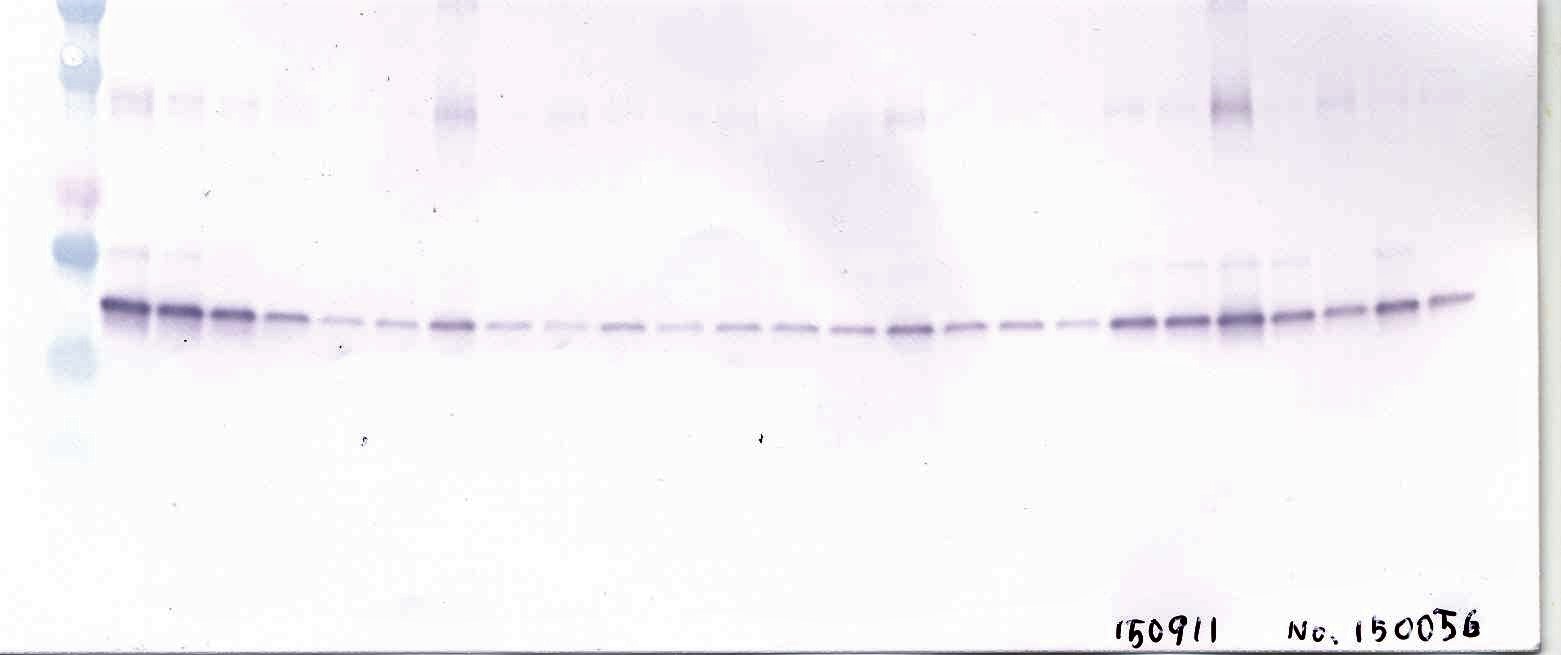

Supplement: Supplementary file 3 — Supplementary Information 3. [file 41598_2022_19491_MOESM3_ESM.jpg]

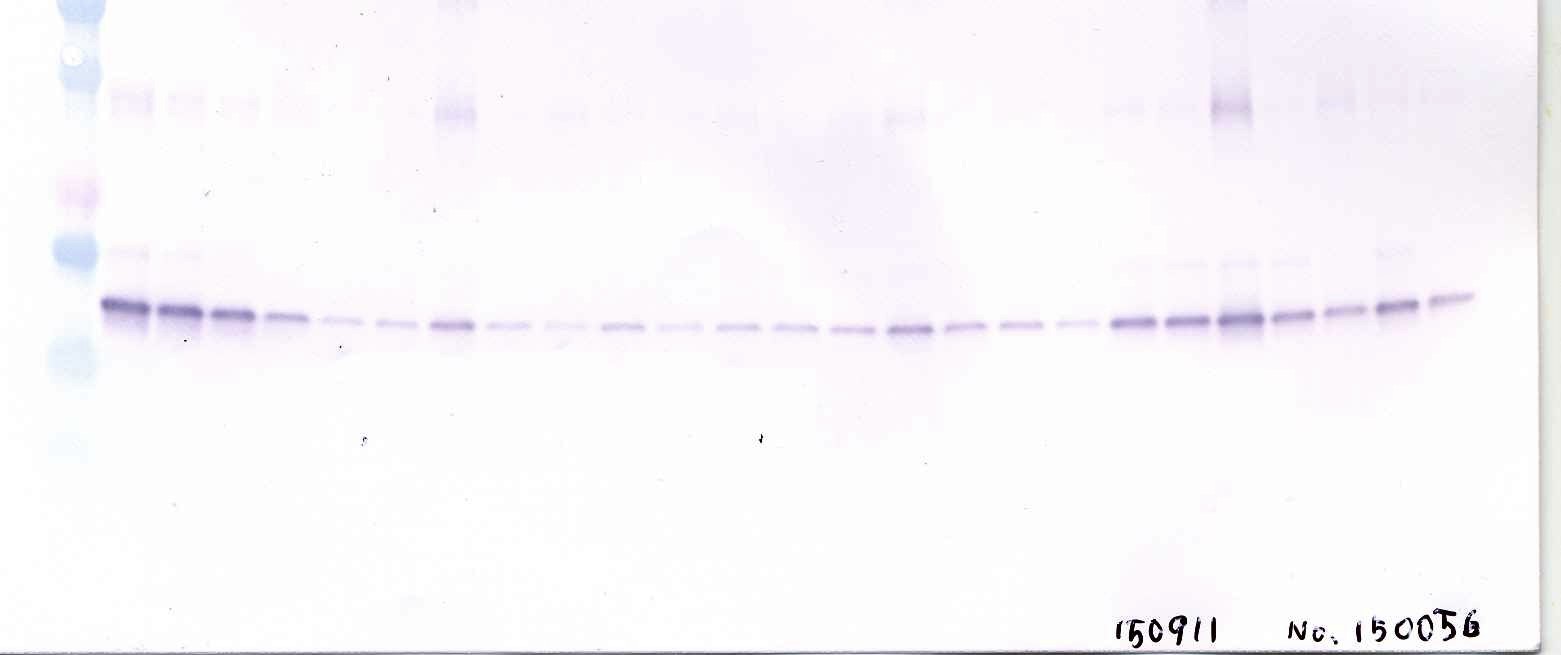

Supplement: Supplementary file 4 — Supplementary Information 4. [file 41598_2022_19491_MOESM4_ESM.jpg]

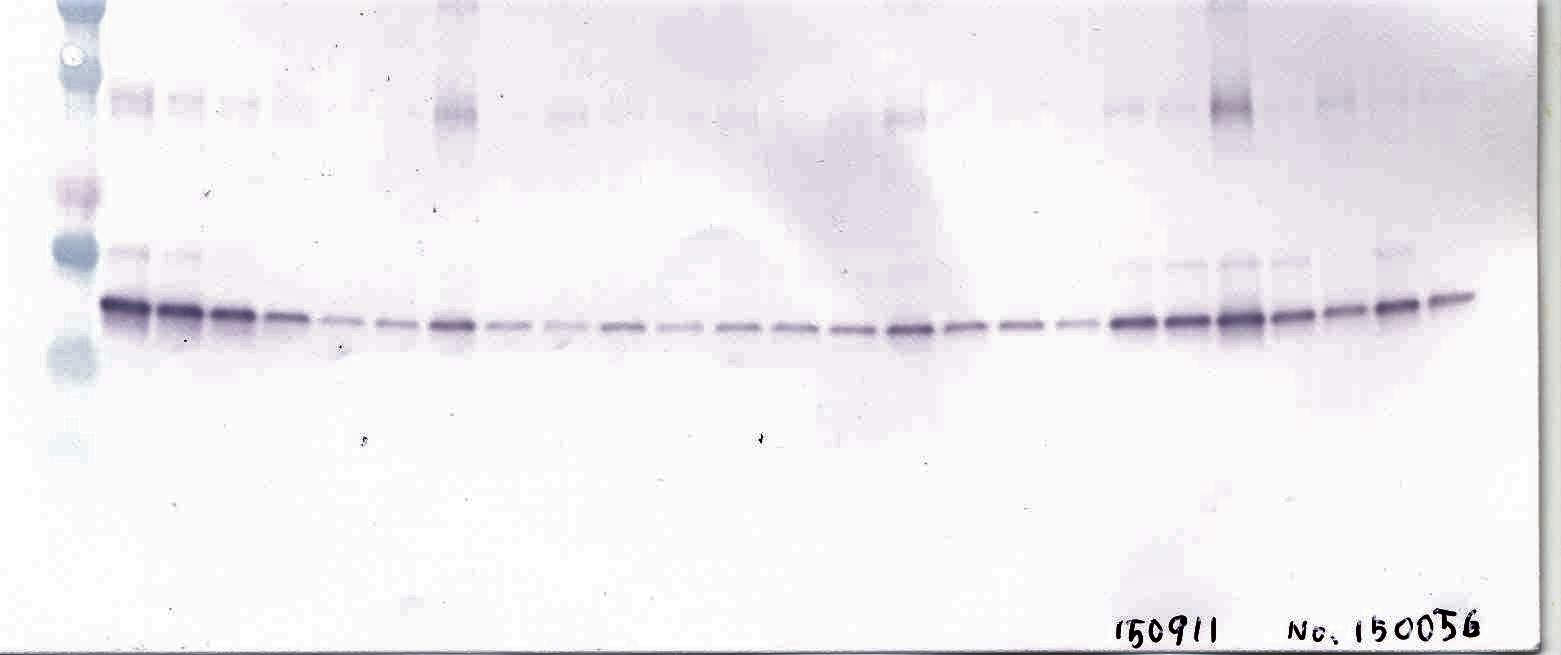

Supplement: Supplementary file 5 — Supplementary Information 5. [file 41598_2022_19491_MOESM5_ESM.jpg]

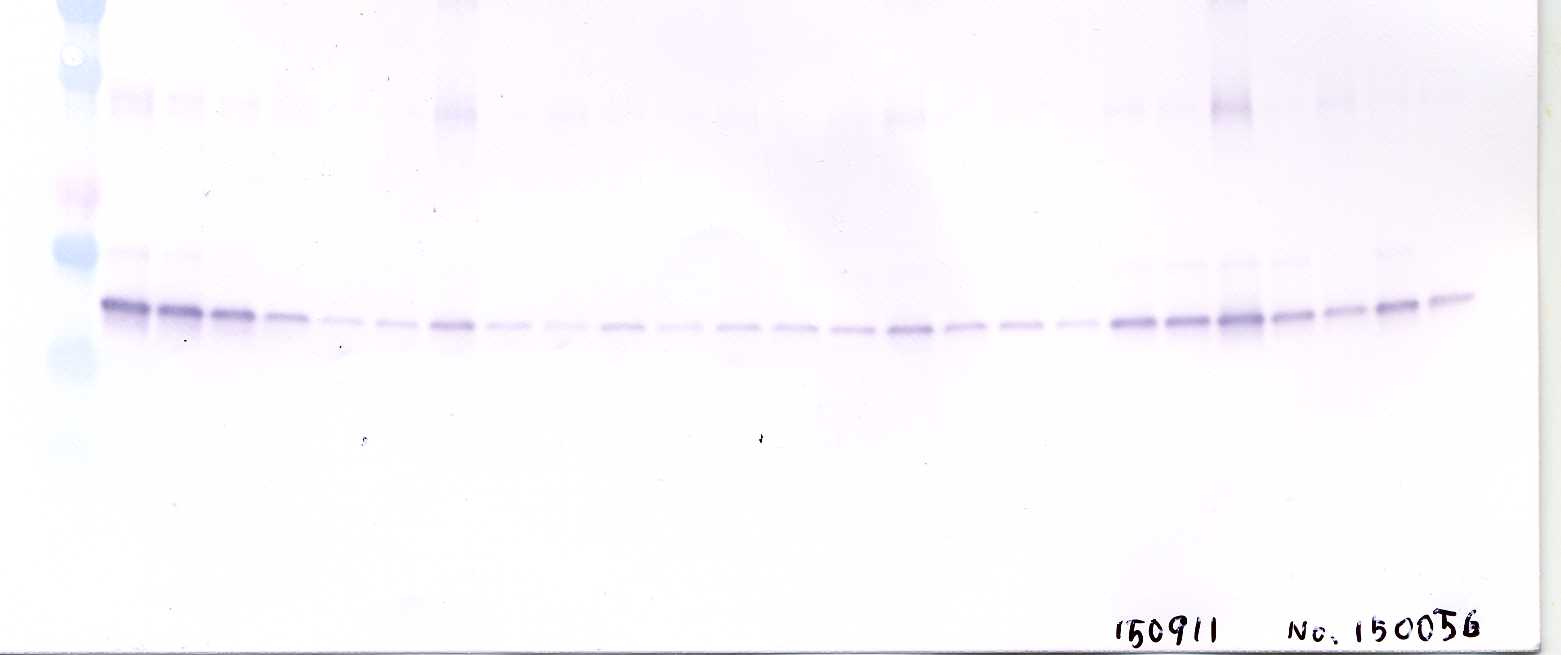

Supplement: Supplementary file 6 — Supplementary Information 6. [file 41598_2022_19491_MOESM6_ESM.jpg]

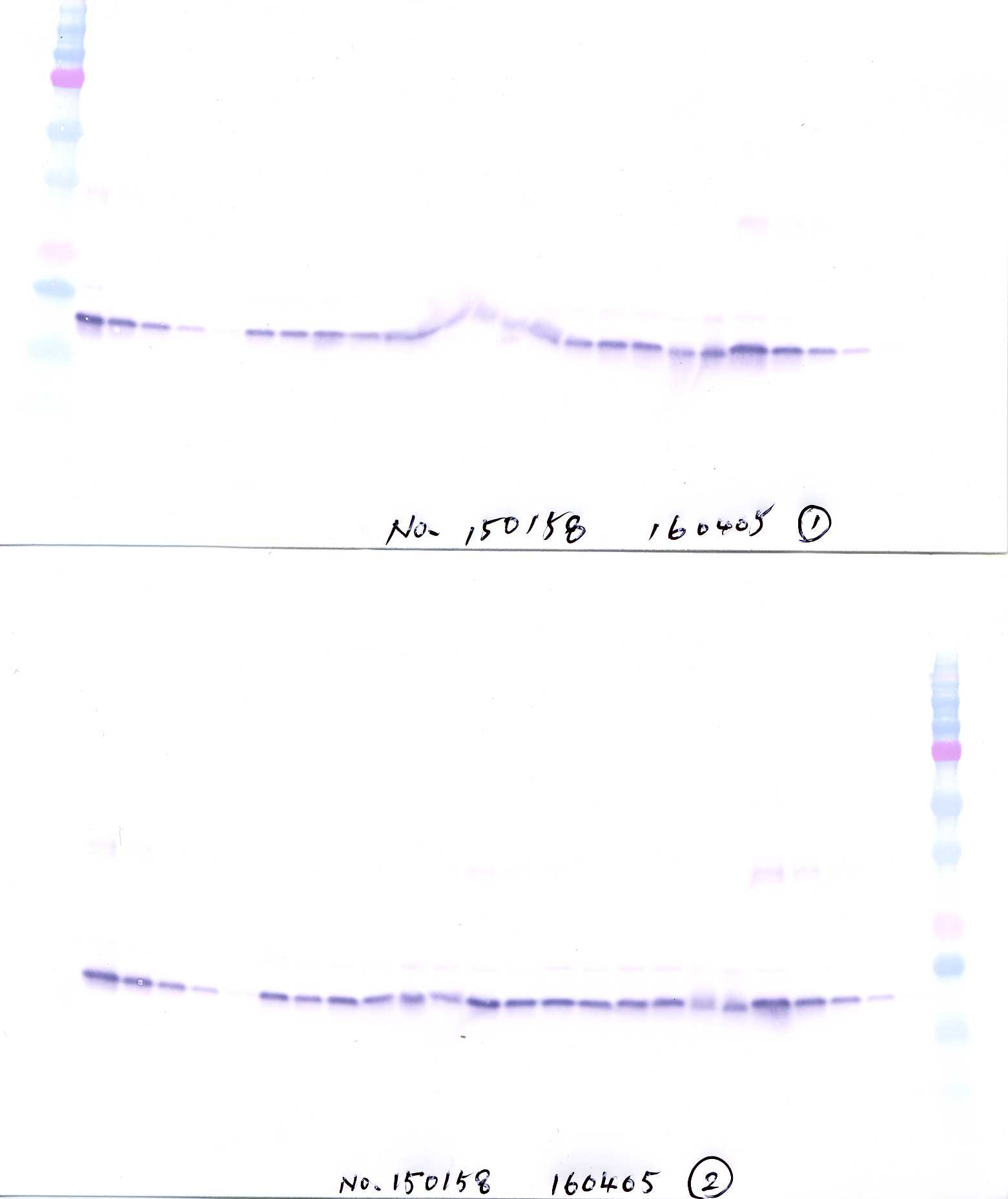

Supplement: Supplementary file 7 — Supplementary Information 7. [file 41598_2022_19491_MOESM7_ESM.jpg]

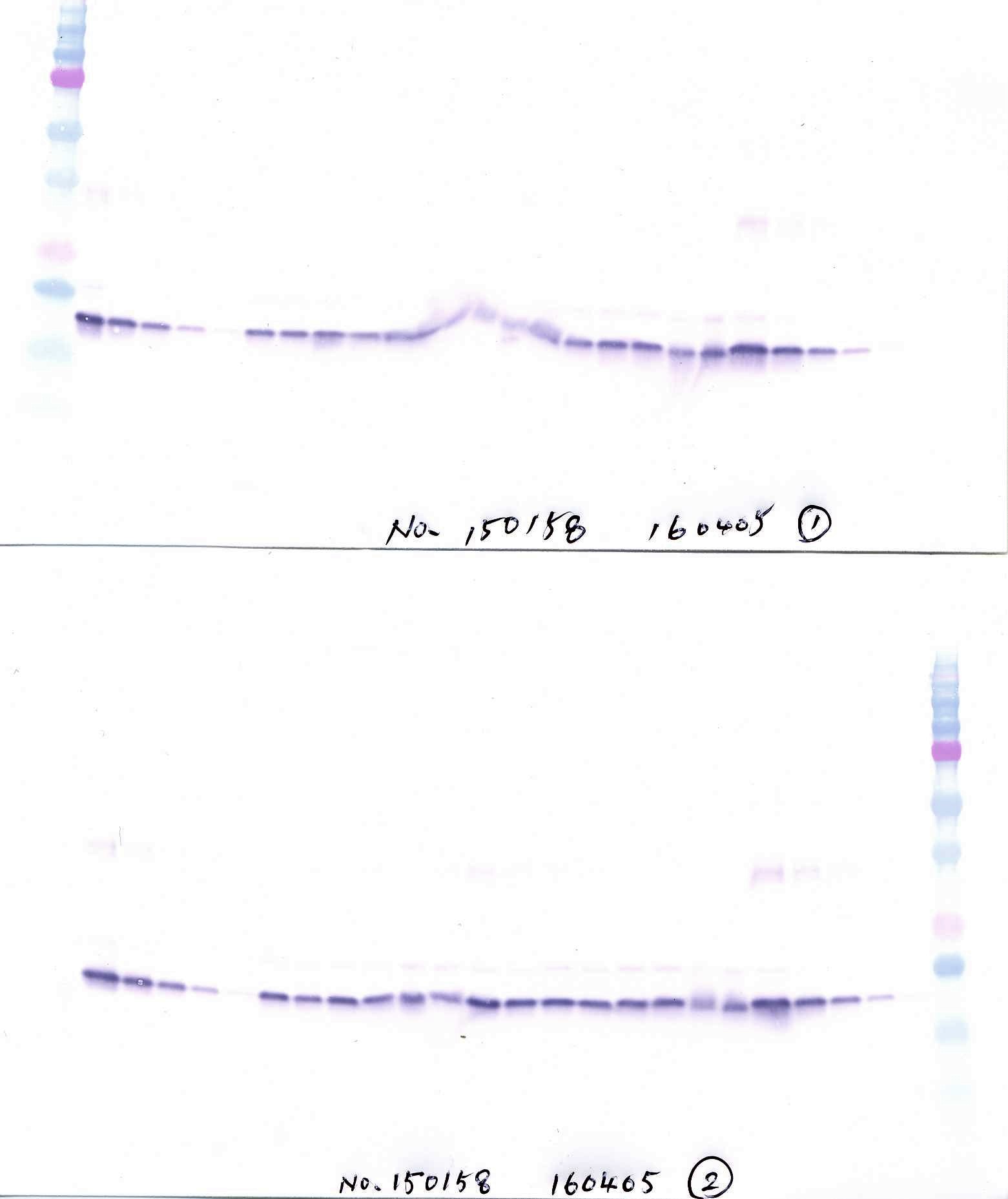

Supplement: Supplementary file 8 — Supplementary Information 8. [file 41598_2022_19491_MOESM8_ESM.jpg]

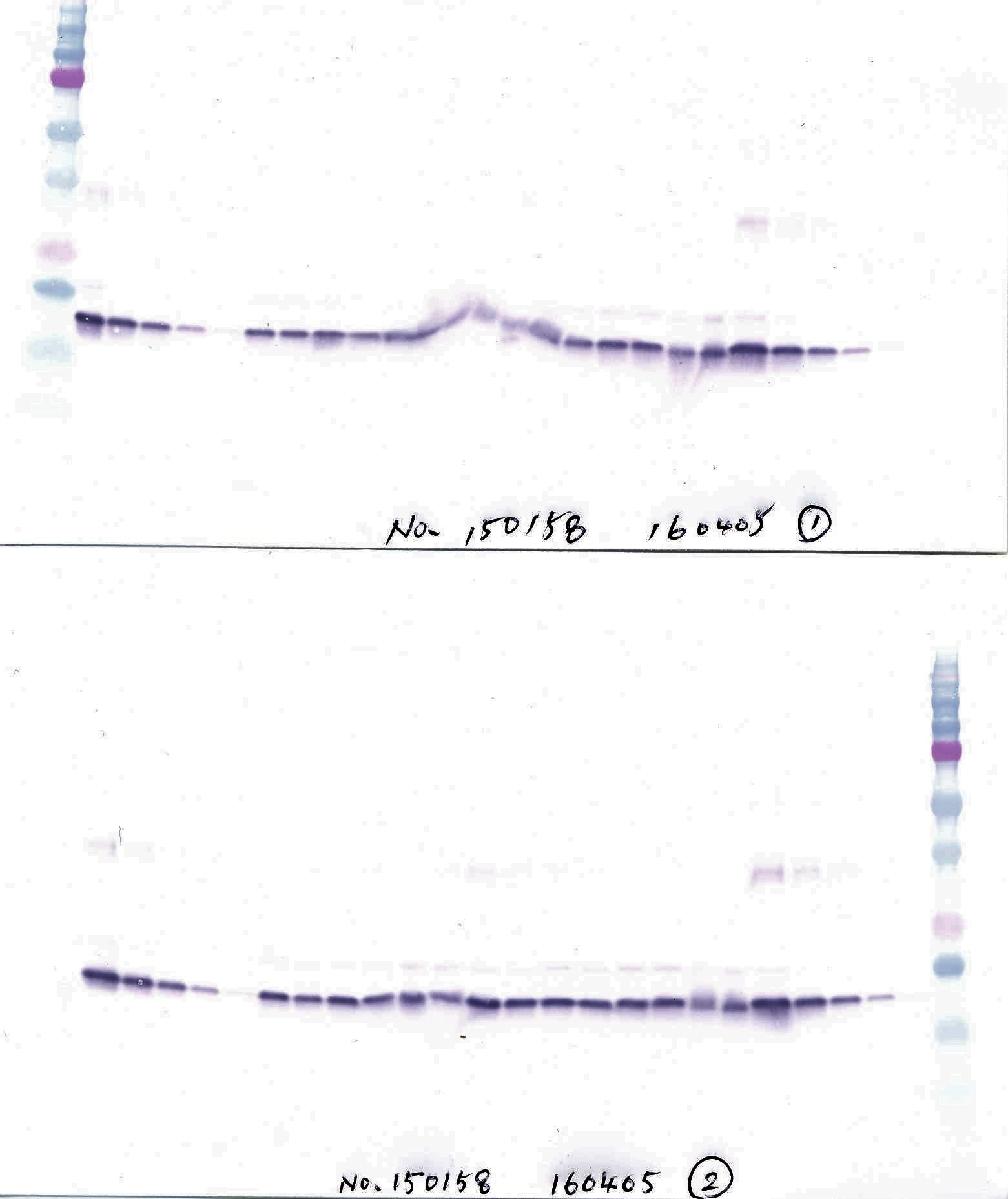

Supplement: Supplementary file 9 — Supplementary Information 9. [file 41598_2022_19491_MOESM9_ESM.jpg]

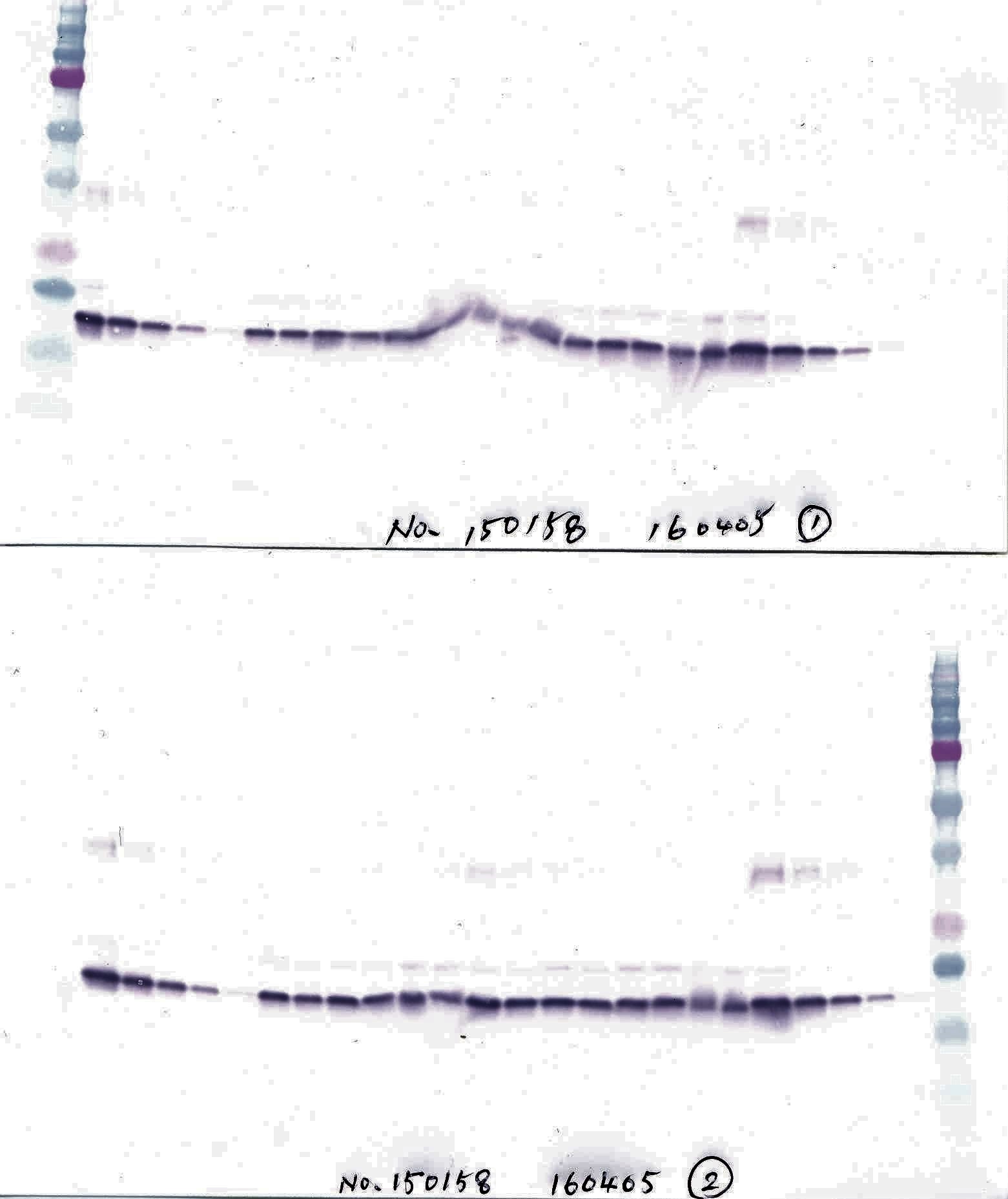

Supplement: Supplementary file 10 — Supplementary Information 10. [file 41598_2022_19491_MOESM10_ESM.jpg]
